# *Bacillus Cereus *Endocarditis in a permanent pacemaker: a case report

**DOI:** 10.1186/1757-1626-1-95

**Published:** 2008-08-18

**Authors:** Salaheldin Abusin, Arvind Bhimaraj, Suhail Khadra

**Affiliations:** 1 Department of Medicine, John H. Stroger Hospital of Cook County, Chicago, IL, 60612, USA; 2 Division of Cardiology, John H. Stroger Hospital of Cook County, Chicago, IL, 60612, USA

## Abstract

**Introduction:**

*Bacillus Cereus *endocarditis is a rare condition which has been implicated in intravenous drug users, and in patients with prosthetic heart valves. We report a rare case of *Bacillus Cereus *infecting a permanent pacing wire.

**Case presentation:**

A 69 year old female with a permanent pacemaker presented with rigors, sweats and weight loss. Blood cultures grew Bacillus Cereus; Transesophageal echocardiogram demonstrated a mobile lesion attached to the pacing wire. She was treated with appropriate intravenous antibiotics for 6 weeks with a good clinical recovery at 6 months follow up.

**Conclusion:**

This case reminds the clinician to have a high index of suspicion for endocarditis in any patient with cardiac prosthesis and to pursue the blood culture results even for rare and unexpected organisms. It also suggests the possibility of a trial of antibiotic therapy prior to prosthesis removal in select patients who are not in heart failure and hemodynamically stable.

## Introduction

*Bacillus Cereus *(BC) is a spore forming bacteria widely distributed in the environment, and as a result when isolated in blood cultures it is sometimes dismissed as a contaminant. It is a significant cause of food poisoning. Non gastrointestinal disease is rare, mainly in the form of local wound infection and ocular infections. It can also cause other infections namely, pneumonia, meningitis, encephalitis, in addition to brain and liver abscesses [1].

It is a rare cause of endocarditis [2] reported in patients with prosthetic heart valves, rheumatic heart disease, intravenous drug use (IVDU), and in a patient with leukaemia.

## Case presentation

A 69 year old lady was admitted to our institution with one month history of shortness of breath, rigors, sweats and significant loss of weight. She had a dual chamber pacemaker fitted in six years prior to this admission. She also suffered from Diabetes Mellitus and Hypertension. On examination, she was afebrile, with stable vital signs, and a 3/6 systolic murmur that was louder with inspiration. A series of peripheral blood cultures were collected, (10 sets in total), and all grew BC.

**Figure 1:**
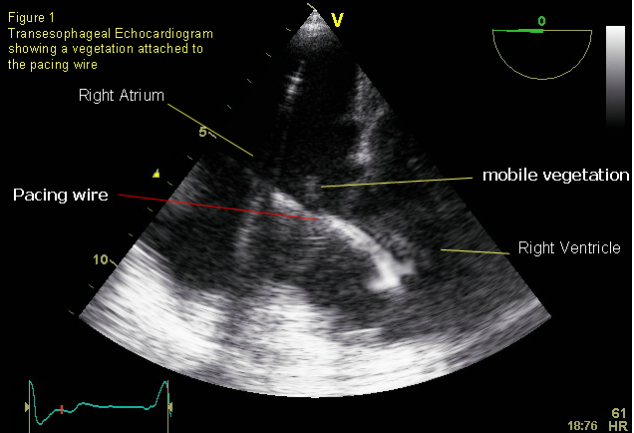
Labelled Transesophagael image.

**Figure 2:**
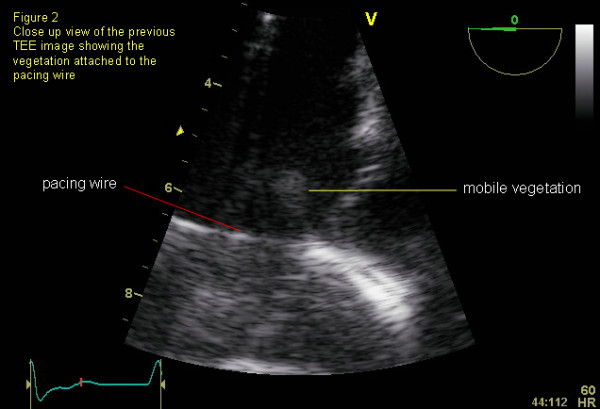
**Labelled Transesophagael image**.

Transesophageal echocardiogram revealed a mobile lesion attached to the pacing wire (Figure 1&2) and moderate tricuspid regurgitation (TR). She was diagnosed with pacemaker associated infective endocarditis. She was known to have TR (secondary to pacemaker lead) and comparison to prior echo showed no significant change.

**Figure 3:**
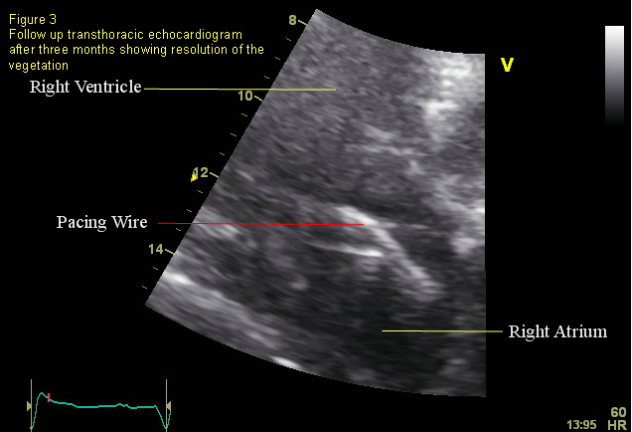
Labelled transthoracic image.

Because the patient was clinically stable, we elected for a trial of intravenous antibiotic therapy prior to surgical removal of the pacemaker. The patient was treated with intravenous Cefazolin, to which the organism was sensitive, for six weeks. She was closely followed after discharge, and at six months follow-up the patient had made a good clinical recovery. A good quality follow-up transthoracic echocardiogram was performed and showed no evidence of vegetation (Figure 3).

## Discussion

There are 14 reported cases of BC Endocarditis in the literature. When it occurs as native valve endocarditis in IVDU patients (6 reported cases), it has an excellent clinical response to antibiotic therapy alone [3-5].

However, in patients with prosthetic heart valves, BC endocarditis has been reported to cause death when treated with antibiotics alone (one reported case) [6]. Surgical replacement of the affected valve is required (4 reported cases) [7-10].

It is also a cause of death in immunocompromised patients and can be difficult to diagnose in this patient population [11] (one reported case at autopsy).

There is one previous case where Bacillus species infected a pacing wire and required removal of the pacemaker [12]. In another report, Bacillus species infected an implantable cardioverter-defibrillator (ICD) lead [13]; the patient was successfully treated with antibiotics alone without the need for removal of the ICD.

In our patient, we collected a large number of blood cultures to refute the possibility of a skin contaminated sample. Although, recent guidelines generally recommend removal of the cardiac prosthesis [14], we elected a trial of antibiotic therapy given the fact that the patient was clinically stable and the peri-operative morbidity associated with the surgical removal of the pacemaker. This approach achieved a good clinical response in our patient.

## Conclusion

This case reminds the clinician to have a high index of suspicion for endocarditis in any patient with cardiac prosthesis, and try to pursue the blood culture results even for rare and unexpected organisms. It also suggests a trial of antibiotic therapy prior to prosthesis removal in selected patients who are not in heart failure and hemodynamically stable.

## Competing interests

The authors declare that they have no competing interests.

## Authors' contributions

SA, was involved in the clinical care of the patient, and contributed to the writing of the manuscript, AB analyzed and interpreted the patient data and was a major contributor to the writing of the manuscript, SK was the cardiology attending in charge of the patient, and was a major contributor to the writing of the manuscript. All authors read and approved the final manuscript.

## Consent

Written informed consent was obtained from the patient for publication of this case report and accompanying images. A copy of the written consent is available for review by the Editor-in-Chief of this journal.
